# Is Dentistry a Specialty of Medicine?

**Published:** 1879-03

**Authors:** C. Stoddard Smith


### ARTICLE III.

### *Is Dentistry a Specialty of Medicine?*

BY C. STODDARD SMITH.

The common, in fact almost universal and generally accepted answer to the question contained in the caption is, that dentistry *is* a specialty of medicine. That such is the case has been assumed by colleges, which embody this idea in their announcements and curriculums; by societies which so state in their constitutions; and by writers, journalists, and practitioners generally.

In this paper we shall take issue with this view of the matter, and shall present such reasons as occur to us in support of the proposition that dentistry *is not*, or at least *ought not to be*, a specialty of medicine.

If dentistry *was* a specialty of medicine, it would follow that the medical text books and curriculums should embrace a more or less complete exposition of dental science; that a medically-educated man would *by virtue* of his medical education and knowledge, be at least measurably fitted to practice dentistry. Are these propositions true? Is either of them true? Let us see.

First, do the medical text books contain, and do the medical professors teach, anything which by any means could be considered an approach to correct dental teachings? It is notorious that they do not, as could be abundantly shown by extracts from standard medical works, which want of space will not permit us to make.

*Incomplete*, as applied to these teachings is not the word; *inaccurate* is better, but does not express the fact; ridiculous nonsense is nearer to it in many cases. These books show that the writers, eminent men in their profession, had not the slightest idea of the true cause of dental troubles, or their appropriate remedies. This is not to be wondered at. It is but a short time since they were, on strictly *medical* subjects, floundering in the depths of ignorance; treating diseases as "humors," blistering, purging and dosing in a wholly empirical manner; and,—the more's the pity,—they have not wholly gotten over it yet. But does this indicate that they are competent to teach dentists what they need to know in order to practice dentistry successfully? Do the teachings or the books in any degree fit the student for such a practice?

Then, second. Is a medically-educated man able, *by virtue of* his medical education, to practice dentistry properly? A moment's reflection will, I think, convince a thoughtful and observant mind that such is not the case. Every one of you *knows* it is not. You know, and I know, that if the preservation of our own teeth, or those of our families, depended upon the treatment they could receive, not from the young medical graduate merely, with the odor of the hospital and dissecting-room still clinging to him, but from the educated and talented physician or surgeon, of large experience and great success, posted in all the literature of the profession, eminent in diagnosis, we should stand but an exceedingly slim chance of retaining any of them longer than Dame nature and the destructive influences of the mouth would allow them to remain. Imagine yourself for a moment, with a carious cavity in close proximity to the pulp, and dependent for treatment upon the village doctor, or even on the most skillful medicus you can call to mind. Do you think you would sit calmly and allow him to scrape and punch that tooth because he was a fine anatomist, or because he had eminent skill in the treatment of typhoid or scarlet fever? In all candor, would you not rather trust the village jeweler, (supposing him to be an intelligent man,) to whom in a half hour's talk and demonstration you could explain the location of the pulp, and the operation necessary? We had almost said would you not rather trust the village blacksmith, or the machinist from the shop? For our own part, we would not only sooner trust the jeweler, but if we wanted to make a successful and a skillful dentist, we would select the intelligent jeweler, or even machinist, in preference to the doctor, and there would be reason in the choice. The training in the one case would have been in the line of the daily requirements of the dentist; in the other it would have been in quite another direction. Medical education, be it ever so thorough does not in any degree qualify, it does not even *prepare* its possessor for dental practice; at least not nearly as much so as does the work of the jeweler, or mathematical instrument maker, who are accustomed to handling delicate instruments and to making fine adjustments. Even as regards the comparatively simple and measurably surgical operation of extracting teeth, do you know any, or at least many general practitioners who perform it with any degree of skill? Do you not have any number of broken teeth coming from them as an evidence of their bungling when they attempt to perform what ordinarily is but a simple operation of what is claimed to be only a "specialty of medicine?" If we are to judge what they know of their profession by what they know or what they can do in what is claimed as a specialty of that profession, they are but a sorry set of men to be intrusted with the life and health of their fellow creatures. I have a better opinion of them than that. I believe their knowledge and ability on *this* subject, is not an index of their skill and success in their own department. They *do* know medicine, but they *don't* know dentistry; and the best of them know they do not. The more intelligent and enlightened they become as regards dentistry, the less they want to meddle with it or its operations, unless indeed they become dentists.

So much for the skill; now for the knowledge. I need but to refer to the oft-told tales of doctors who treat alveolar abscess for months supposing it to be erysipelas, who treat neuralgia as a constitutional disease, because the "teeth are all sound," or have fine "solid" fillings in them; who do not know that a wisdom-tooth may cause almost any trouble about the face; of the surgeons who gravely pronounce an old root covered with salivary calculus, to be an "osteosarcoma;" of the almost universal practice of the M. D.'s who prescribe acid medicaments in blissful ignorance or willful disregard of their effect upon the dental structures; of the doctor who assures the parent that the sixth-year molar is a milk tooth, and should be extracted. Every one of us has seen more or less of this sort of thing; every one of us knows that these accounts are usually accompanied by the statement that the thing was "done by one of our *best* physicians." These things show, not only that medical men, *as such*, have no *skill* in dentistry, but that they are wofully deficient in *knowledge* as well; in fact they are but little above the intelligent non-professional in either respect.

And further; do medical men necessarily or even usually make the most successful or skillful dentists? We will not say what has been said, *M. D.* stands for miserable dentist; but we will say that in our opinion, as a rule the *M. D.* members of the profession are not at least any better than the rest; and we do not believe they will average in ability as well as an equal number of equally intelligent non-medical men. Call to mind those of your acquaintance and see how they stand. Go abroad and see how the long, scholastic and medical European training makes fine operators, or rather see how it does *not* do it.

The main reason, as we understand it, for claiming that dentistry is a speciality of medicine, is that the teeth are a part of the human frame; that they and the adjacent parts are subject to disease; and that he who treats those diseases properly must understand the human frame, and the treatment of disease; *ergo* he is a physician. Indeed it has been broadly stated that if we are not medical specialists we are a set of carpenters. But let us see if this statement is really true—if this conclusion necessarily follows. Granted that the teeth are a part of the human organism, and subject to disease, which none deny. Granted that a knowledge of anatomy, of physiology, of therapeutics, is necessary to the proper treatment of dental lesions. Does it follow because the medical man must also study these—because both he and the dentist are obliged to get a part of their preliminary information from the same text books—because certain knowledge underlies both professions, that the one is a branch or specialty of the other? All knowledge is founded upon certain substructures which are common to all branches alike. What sort of an argument would it be to say that architecture was a branch or specialty of astronomy, because both the architect and the astronomer must understand mathematics, and must occasionally use the rule of three in working out their problems; because both make drawings upon paper to record the work of their brains? Shall we say that pharmacy is a specialty of medicine because both require a knowledge of drugs and chemicals? Shall we say that the maker of artificial legs is a medical specialist, because he would need to understand the anatomy of the leg in order to construct his substitute, and because he has to deal with living tissue when applying it? The temple of science is not a collection of columns, each standing upon its own pedestal, and each crowned with its appropriate bust or sculpture. It is rather a magnificent edifice, whose foundation stones are planted upon the solid rock of truth, and are interlaced and interlocked; its lower stories are all communicating, and all subservient to the uses of the upper parts, from which rise the several spires, cupolas, turrets, minarets and towers devoted to the various branches of science and art, differing, it may be, in architecture, in height, in magnificence, but all alike parts of one harmonious and imposing whole.

It is true that the dentist and the physician must have much knowledge in common; but it is not therefore true that dentistry is a branch of medicine. Though there is much in common, there is more that is not so. What has the physician (as such) to do with metallurgy and plaster-of-paris, and sand and zinc, and tempering steel; with the cohesive properties of gold, the proper vulcanizing point of rubber, or the manipulation of celluloid? What does he know,—what in the nature of the case *can* he know, about the thousand delicate manipulations of the skillful dentist? His time and brain are full of symptoms and doses; if he knew ever so much dentistry he would not be able to use his knowledge. What has the dentist (as such) to do with obstetrics, with ophthalmology, with cardiac disease, with lung or liver disease, with cerebral disease, with venereal disease? Why should he waste his time, and that to no purpose, in studying these diseases, their symptoms and their treatment? We say waste his time, not because such knowledge is not desirable, and might not be valuable; so might a knowledge of chemistry, or of many other things; but who expects a dentist to be a thorough chemist, and how is it possible for him to be one? Chemistry is not a by-play, a thing to be mastered in odd moments; it is a life-study, and enough so to engage the ablest minds. We say waste his time, because all the knowledge that he might obtain from books on these subjects, or even from a hospital experience, should he have it, would be utterly useless to him in his life work; not because he might not have occasion to use it, but because when wanted he could not depend on it. Such knowledge, to be of any practical use, demands not only preliminary study and clinical experience, but constant and ceaseless observation and practice, in order to render it of any reliable service, which, by the nature of the case, is out of the question. Which of you, if you filled a tooth but once a year or once in five years, could do justice to the work? What graduate (unless he be possessed of an exceptional memory,) can tell three years after commencement the names of the muscles, or the bones, or the origin and function of the nervous trunks? Who of you would trust the life of a dear one in a crisis, with a man who, though he might *know* the symptoms of the fever, was not practically familiar with the thing itself?

We say waste his time, not because there is not much in these things that is desirable and proper for a dentist to know, but because it is utterly impossible that he should know all or even any considerable portion of what is just as desirable and proper to be known. Dentistry itself is a life-work, a life-study; it takes a whole man to be a dentist, and it should occupy the whole attention. For a man to undertake to thoroughly master all that is collateral to it, would be like the attempt of the landshark to buy all the land that joined him. He would soon find himself upon a limitless expanse. The thing is impossible; it cannot be done. To attempt it would be to frustrate the objects aimed at, for no time or strength would be left for the thing itself. And when it comes to choosing, as it certainly must come, it is the part of wisdom to choose that which will best fit one for the practical realities of one's chosen pursuit. In ordinary education this is being more and more realized; it is recognized that the study of the classics and other branches, do not tend to fit men,—indeed it is sometimes claimed that they *unfit* them—for the practical business of life; and consequently, technical and polytechnic and other practical courses of study are more popular than classical. The ideal dentist may be fully educated in chemistry, in metallurgy, in physiology, in therapeutics, in anatomy, and all kindred and cognate sciences; but that ideal dentist will never exist while flesh and blood and human capacity remain as they now are, simply because his existence would involve physical impossibilities.

Let us not be misunderstood. We go for the broadest, the highest, the deepest culture possible, in all departments of human activity. But life is short and art is long. It is given to but few men to become possessed of eminent or even of tolerable knowledge in more than one department; it is, in fact, given to but few to become really eminent in even one department. The great mass must rest content if they attain sufficient knowledge to enable them to become efficient workers in any single department of the world's great work-shop. But let every man *first* possess himself of just as much preliminary education as his position, means, and ambition will enable him to get; then, having settled upon his calling, master that; *afterwards*, let him acquire just as much collateral or other knowledge, as his situation and justice to his chosen occupation will permit. But let him not fritter away his energies in an attempt to make a quart measure hold a peck, or a blanket cover an acre; to master all the branches of medicine because he wishes to be dubbed a specialist.

Far better will it be, both for him and his patients (if he is to be a dentist,) that he spend his time and powers in obtaining that skill which he will need every day, and of which there is no danger of his having too much.

But it is said that the greater includes the less, and that anything else than a full M. D. is but a "partial culture."

The greater does include the less; but who said that dentistry was less than medicine? That is assuming the very point under discussion; arguing in a circle. Those who claim that dentistry is a specialty of medicine, would make it *less; we* claim that it is the proud *peer* of medical science. Not in age, it is true; we cannot look down the vistas of the past and see an unbroken succession from the times of Galen and Esculapius. But in our vigorous young manhood, in the skill and certainty we have attained in the performance of our operations, in the "good that we can do" to suffering humanity, palliative, remedial and prophylactic, we are certainly the peers of any craft, profession or calling, be it ancient or modern, be it professional or mechanical. We are *not* "the less," nor are we included in the greater.

"Anything short of a full M. D. is a 'partial culture.'" We have shown in what seems to us an unanswerable argument, that an M. D. does not imply, or even indicate, a capacity to practice dentistry. As to the statement, that an education which fits a man only to practice dentistry, is but a "partial culture;" we admit it; it *is* but a "partial culture." But show us the inhabitant of this sublunary sphere, who has anything else than a partial culture. What title conferred by mortal man, indicates that its professor has mastered all that is to be known; that no worlds in the realms of knowledge remain for him to conquer; that all the cosmogony and theology, of the here and the hereafter, is to him but as a lesson learned? A "partial culture," it is true; but alas for human nature; it must await translation to another sphere, where knowledge and existence are alike infinite, before it can hope for more than this. A "partial culture;" yes; but Newton, the great and erudite philosopher, to whose acquirements, few if any, can aspire, much less attain, was forced to admit at the close of his life, that so far from crossing the great ocean of knowledge, all that he had done was to stroll along its shores and gather a few pebbles from the beach.

The fact is, and it cannot be disputed, that nine tenths of the practice of dentistry, is mechanical. It is worthy of notice, that the more completely we accomplish that thing in regard to which we talk so much, and we fear accomplish so little—the enlightenment of the public—the more complete the mechanical character of the practice becomes; that is, the better care people can be induced to take of their teeth: the oftener they submit themselves and their children for treatment, the less of disease as such, as distinguished from dental caries, we will have to take cognizance of, and the simpler our operations as a whole will become. If dental caries is a disease at all, the treatment of it (simple caries of course, we mean) is purely mechanical. And sometimes we think that with all our boasted progress, we who can circumnavigate the globe, can put a girdle around the earth in forty minutes, can tunnel under lakes, and rivers, and mountains, can talk through wires, and make the lifeless iron speak, who can count the very stars of the heavens, and weigh the sun as in a balance; ought to be ashamed of ourselves when we are compelled to admit that we cannot put a gold filling into a little miserable cavity in a tooth, so that it will arrest decay, or find something else that will do it.

But dental practice is not *all* mechanical; else it were a trade as is the tinker's, as it was when it began, or as is the barber's trade to-day. It requires with this mechanical dexterity, education of the brain, to know the characteristics of the parts on which it operates; it requires judgment study and thought all the way through, and these constitute it a profession. But it is a pursuit *sui generis*, of its own exclusive kind, and as I maintain, separate and distinct from all the rest. An education of the fingers far exceeding that of most of the trades; education of the head as well as the hand; judgment, skill, dexterity, and all of the highest order; are not these qualities sufficient to give us a foundation upon which to rear our own edifice, without asking to be attached like a lean-to to the great temple of

Esculapius? Do we gain dignity by clinging to the coat-tails of medicine, clamoring for "recognition," instead of standing boldly up and proclaiming ourselves, as did the infant colonies, free and independent? Away with such servile cringing; such degrading sycophancy! *They* do not want us within their doors; let us manfully stay outside, and show them that while we understand *our* business, we do not claim to understand theirs. Let us show them that we mean to be *dentists*, and not medical specialists. Let us know enough of the branches common to both professions, to enable us to consult with them intelligently; enough of their profession to know when we should hand our patients over to them. Let us respect their rights and knowledge in their department, as we ask them to respect ours, and not interfere with their pills and powders, or undertake to treat teething children or miasmatic influences. Let us ask them to study so much of our professional knowledge, as to know enough when anything is the matter in the region of the mouth, to consult an intelligent and skillful dentist, and be willing to abide by his advice; and let us during such intercourse, not endeavor to create the impression upon the mind of either the physician or patient, that we are physicians, which we are not, and understand medicine, which from the nature of the case we cannot; but rather let us show by our conversation and opinions, but above all, by our operations, that we are *dentists* and understand *dentistry*. Let each profession know thoroughly so far as necessary to each, those branches which underlie both in common; let us know in addition, all we can of the other's profession, as we do of all other knowledge which goes to make us intelligent, educated and well informed citizens. Let us make both the medical profession and the community respect our skill and attainments; but do not let us lay claim to being what we are not. Thus, side by side, and hand in hand, let the two sister professions go together, each according to the other, all the honor, dignity and knowledge to which each is entitled; fraternizing and consulting with each other and laboring together, and in unison, for the good of our fellow men.—*Proceedings of Ill. State Dental Society.*

---

### ARTICLE IV.

### *The Virginia State Dental Association.*

